# The Book of Prescriptions

**Published:** 1864-11

**Authors:** 


For sale by W. B. Keen & Co., 148 Lake Street, Chicago.

---

THE BOOK OF PRESCRIPTIONS, containing 3000 prescriptions, collected from the practice of the most eminent Physicians and Surgeons, English, French, and American. Comprising also a Compendious History of the Materia Medica; Lists of the Doses of all Official or Established Preparations; and an Index of Diseases and Remedies. By HENRY BEASLEY, Author of the "Druggist's Receipt Book," and the "Medical Formulary." Philadelphia: LINDSAY & BLAKISTON. 1864.

This is a full-sized octavo volume of about 300 pages, and is probably the most useful book of its kind, accessible to the student. The contents of the book are fairly indicated by the title-page.

